# Caldesmon: Biochemical and Clinical Implications in Cancer

**DOI:** 10.3389/fcell.2021.634759

**Published:** 2021-02-18

**Authors:** Yi-Bo Yao, Chang-Fang Xiao, Jin-Gen Lu, Chen Wang

**Affiliations:** ^1^Department of Anorectal Surgery, Longhua Hospital, Shanghai University of Traditional Chinese Medicine, Shanghai, China; ^2^Longhua Hospital, Institute of Chinese Traditional Surgery, Shanghai University of Traditional Chinese Medicine, Shanghai, China

**Keywords:** actin binding protein, caldesmon, isoform, smooth muscle, cancer

## Abstract

Caldesmon, an actin-binding protein, can inhibit myosin binding to actin and regulate smooth muscle contraction and relaxation. However, caldesmon has recently attracted attention due to its importance in cancer. The upregulation of caldesmon in several solid cancer tissues has been reported. Caldesmon, as well as its two isoforms, is considered as a biomarker for cancer and a potent suppressor of cancer cell invasion by regulating podosome/invadopodium formation. Therefore, caldesmon may be a promising therapeutic target for diseases such as cancer. Here, we review new studies on the gene transcription, isoform structure, expression, and phosphorylation regulation of caldesmon and discuss its clinical implications in cancer.

## Introduction

Caldesmon, an actin-binding protein of 150 kDa, was first isolated and purified from chicken gizzard muscle in 1981 (Sobue et al., 1981). Caldesmon was named from a combination (desmos is a Greek word that means binding) of calmodulin due to its ability to bind with calmodulin at different Ca^2+^ concentrations (Sobue et al., 1981). Caldesmon has two different molecular weight isoforms: high-molecular-weight caldesmon (H-caldesmon, 120–150 kDa) found in smooth muscle and low-molecular-weight caldesmon (L-caldesmon, 70–80 kDa) found in non-muscle cells (Hayashi et al., 1991; Mayanagi and Sobue, 2011). By cloning and sequencing the cDNA, H-caldesmon and L-caldesmon were determined to be derived by alternative splicing from a single gene (Hayashi et al., 1991). H-caldesmon and L-caldesmon conserve completely identical sequences in the N- and C-terminal domains, and the central repeating sequence of H-caldesmon is deleted in L-caldesmon (Hayashi et al., 1991). Although H-caldesmon and L-caldesmon have similar functional domains, their tissue and cell distributions are distinct (Ball and Kovala, 1988; Sobue et al., 1988).

Caldesmon has recently attracted attention due to its roles in cancer (Mayanagi and Sobue, 2011). Caldesmon can be a biomarker for the pathological diagnosis of tumors and prediction of the chemoradiotherapy response. H-caldesmon is considered a specific marker for tumor with smooth muscle differentiation (Watanabe et al., 1999; Nucci et al., 2001). L-caldesmon-positive human colon cancer cell lines are more resistant to chemoradiotherapy than L-caldesmon-negative cell lines (Kim et al., 2012). Second, caldesmon can also suppress cancer metastasis by regulating the podosome/invadopodium formation in transformed cancer cells, and the suppressive effect has been verified in a variety of cancers (Yoshio et al., 2007). In prostate cancer cells, a twofold increase in migratory capability and a threefold increase in invasion capability were found by scratch and invasion assays after the knockdown of L-caldesmon expression (Dierks et al., 2015). In addition, caldesmon can reversibly and cooperatively inhibit myosin binding actin to regulate smooth muscle contraction (Sobue et al., 1982; Ngai and Walsh, 1984). The phosphorylation of caldesmon plays an important role in the regulation of smooth muscle contraction (Huang et al., 2003). Therefore, this review analyzes the gene transcription, isoform structure, expression, and phosphorylation regulation of caldesmon and its clinical implications in cancer and gastrointestinal motility disorders.

## Caldesmon Gene, Structure, and Expression

**FIGURE 1:**
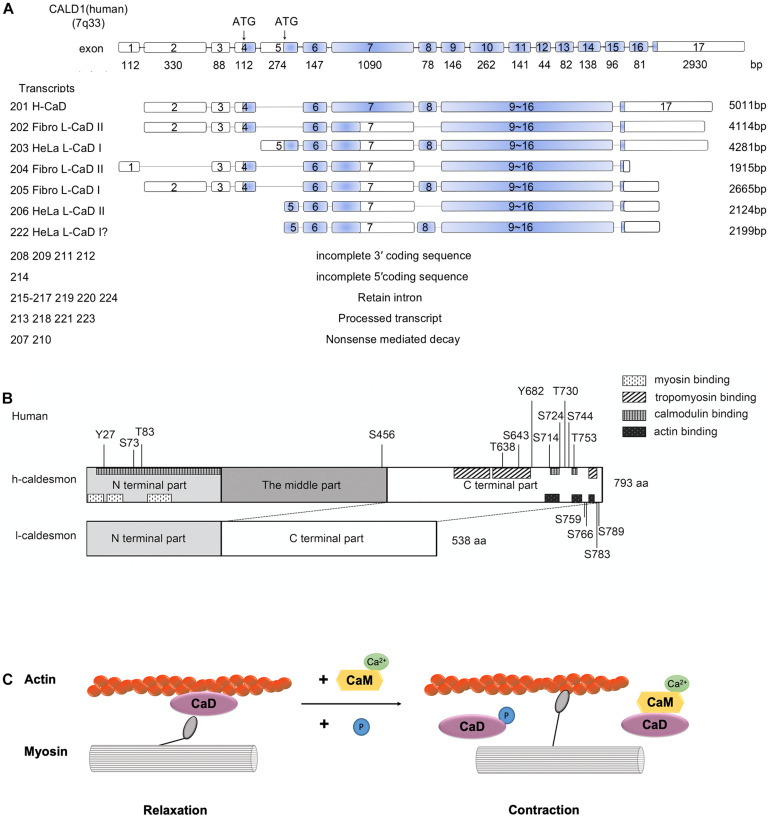
**(A)** Human Caldesmon Gene and Transcripts. Caldesmon (*CALD1*) structure showing exon (numbered boxes) and intron (line) regions and sizes. The caldesmon gene has 24 transcripts (201–224). Transcripts 201–206 and 222 are shown with the translated regions, and the blue regions are the coding sequences of the transcripts. The other transcripts cannot generate caldesmon due to the incomplete 3′/5′ coding sequence, retained introns, processed transcripts, or nonsense-mediated decay. Caldesmon isoforms are mainly generated by the selective spliced exons (exons 7 and 8) and distinct promoters (starting with exon 4 or 5). Exon 4 encodes the N-terminal domains of Fibro L-caldesmon and exon 5 encodes HeLa L-caldesmon. Exon 8 encodes 26 amino acids, including an extension of the α-helical motif. Transcripts 202–206 and 222 can generate Fibro-type and HeLa-type L-caldesmon, transcript 205 (Fibro L-caldesmonI, 563aa), transcript 202 and transcript 204 (Fibro L-caldesmonII, 538aa), transcript 222 and transcript 203 (HeLa L-caldesmonI (557–558aa), and transcript 206 (HeLa L-caldesmonII 532aa). Of these, transcript 222 has not been reported previously and was identified by a database search of Ensembl. **(B)** The domain structures of H-caldesmon and L-caldesmon. Human caldesmon contains an N-terminal domain, a C-terminal domain, and a middle part (repeating domain). The difference between L-caldesmon and H-caldesmon is the deletion of the repeating domain due to alternative splicing. The N-terminal domain contains a myosin-binding site and interacts weakly with actin and calmodulin. The C-terminal part contains an actin-binding site, calmodulin-binding site, tropomyosin-binding site, and phosphorylation sites. Human H-caldesmon (793 aa) is regulated by phosphorylation at Tyr-27, Ser-73, Thr-83, Ser-456, Thr-638, Ser-643, Tyr-682, Ser-714, Ser-724, Thr-730, Ser-744, Thr-753, Ser-759, Ser-766, Ser-783, and Ser-789 through multiple kinases (Cdc2,PAK,PKC, CamKII, CKII, and *v-erbB* tyrosine kinase). **(C)** The role of caldesmon in smooth-muscle contraction. The mechanism of reversing the putative inhibition by caldesmon of smooth muscle contraction by caldesmon depends on Ca^2+^/calmodulin and phosphorylation. Caldesmon can bind to actin filaments at less than 1 μM free Ca^2+^, whereas at a higher concentration of Ca^2+^ (>1 μM), calmodulin activated by Ca^2+^ forms a complex with caldesmon, and this complex is freed from actin filaments. Phosphorylation of caldesmon can attenuate its inhibitory activity, allowing actomyosin interaction and thereby resulting in muscle contraction.

The caldesmon gene is located on human chromosome 7q33 (Ensembl ID of the human caldesmon gene is ENSG00000122786) (Yates et al., 2020; Figure 1A). The caldesmon gene has 17 exons, and its isoforms (H-caldesmon and L-caldesmon) are mainly generated by the selective splicing of exons 7 and 8 (Lin et al., 2009). Exon 7 of selective translation encodes the central repeating sequence, and this central repeating sequence is specific to H-caldesmon (Transcripts 201,793 aa) (Mayanagi and Sobue, 2011). The caldesmon gene has 24 transcripts (201–224). Transcript 201 can generate H-caldesmon, while transcripts 202–206 and 222 can generate L-caldesmon. According to the different promoters, L-caldesmon can be further classified as a Fibro-type (WI-38) or HeLa-type (Hayashi et al., 1991). Two different distinct promoters are used in different cell types or tissues to generate L-caldesmon isoforms with distinct N-terminal domains (Yano et al., 1994). Alternative splicing of the caldesmon gene determines the different structures and expression of isoforms (Hayashi et al., 1991).

From a structural perspective, caldesmon contains amino (N)- and carboxy (C)-terminal domains and a middle region (Wang, 2001). The N-terminal part can bind myosin and calmodulin (Lin et al., 2009); the C-terminal part contains actin-binding sites, calmodulin sites, and tropomyosin-binding sites (Wang, 2001; Mayanagi and Sobue, 2011), and the middle region in H-caldesmon (208–462 aa in humans) contains a long α-helix region and separates the N-terminal domain from the C-terminal domain (Wang, 2008; Lin et al., 2009; Figure 1B). The middle region is only present in H-caldesmon and is missing in L-caldesmon due to alternative splicing (Hayashi et al., 1991; Mayanagi and Sobue, 2011). However, the function of the middle region remains unknown. The middle region in H-caldesmon is presumed to fit the specific spatial arrangement of myosin molecules in the smooth muscle thick filament by evolutionary optimization (Wang, 2008).

The tissue and cell distributions of H-caldesmon and L-caldesmon are different. H-caldesmon is expressed in vascular and visceral smooth muscle and not in myofibroblasts, rhabdomyosarcoma, or tumors derived from myofibroblasts (Rush et al., 2001; Fisher et al., 2003). Therefore, H-caldesmon, as a smooth muscle-specific biomarker, can distinguish tumors originating from smooth muscle. In contrast, L-caldesmon is widely distributed in non-muscle tissues, such as the brain, spleen, and lymph nodes (Köhler, 2010, 2011). However, the expression changes of the two isoforms are closely correlated with the phenotypic modulation of smooth muscle cells (Ueki et al., 1987; Yokouchi et al., 2006). The expression of caldesmon can switch from L-caldesmon to H-caldesmon during smooth muscle cell differentiation and the expression turns from H-caldesmon to L-caldesmon during the dedifferentiation of smooth muscle cells (Ueki et al., 1987). Therefore, the different expressional distributions determine the different functions of H-caldesmon and L-caldesmon.

## Post-Translational Regulation of Caldesmon

Caldesmon is an actin, myosin, tropomyosin, and Ca^2+^/calmodulin binding protein capable of regulating actomyosin contraction, actin filament dynamics, and cytoskeleton remodeling in smooth muscle and non-muscle cells (Lin et al., 2009). Posttranslational modification of caldesmon can modify its function and has been studied extensively *in vitro* (Foster et al., 2004; Ng et al., 2018). The association of caldesmon with tropomyosin-containing actin filaments effectively inhibits actomyosin ATPase activity and *in vitro* actin filament motility (Lin et al., 2009).

The mechanism of reversing the putative inhibition by caldesmon of smooth muscle contraction by caldesmon depends on Ca^2+^/calmodulin and phosphorylation (Foster et al., 2004; Mayanagi and Sobue, 2011; Figure 1C). Depending on the concentration of Ca^2+^, caldesmon shows an alternative binding ability to either calmodulin or actin filaments *in vitro* (Sobue et al., 1981). Caldesmon can bind to actin filaments at less than 1 μM free Ca^2+^, whereas at a higher concentration of Ca^2+^ (>1 μM), calmodulin activated by Ca^2+^ forms a complex with caldesmon, and this complex is freed from actin filaments (Sobue et al., 1981).

An alternative mechanism calls for phosphorylation of caldesmon in view of the fact that smooth muscles can contract at low Ca^2+^ concentrations (Foster et al., 2004). The phosphorylation of caldesmon is closely related to smooth muscle contraction (Hai and Gu, 2006). In an *in vitro* motility assay, unphosphorylated myosin exerted a mechanical load to shorten filaments, suggesting that tethering thick and thin filaments by caldesmon might help maintain some basal force (Horiuchi and Chacko, 1995). Phosphorylation (such as Thr-627, Ser-631, Ser-635, and Ser-642) can attenuate the inhibitory activity of caldesmon and indirectly increase inhibitory activity by weakening binding to Ca^2+^-calmodulin (Hamden et al., 2010). The interplay between phosphorylation-dependent and Ca^2+^/calmodulin-dependent mechanisms may be complex. The effect of Ca^2+^/calmodulin on the activity of caldesmon is dependent on the combination of phosphorylated residues (Hamden et al., 2010).

As a downstream effector of multiple signaling pathways, the inhibition of caldesmon can be reversed by phosphorylation during smooth muscle contraction through multiple kinases, such as ERK and PAK (Hai and Gu, 2006; Lin et al., 2009). Extracellular regulated kinase (ERK)-mediated phosphorylation of caldesmon has been shown to reverse the ability of the actin-binding fragment of caldesmon to stabilize actin filaments (Hai and Gu, 2006). Phosphorylation of caldesmon at ERK sites (Ser-759 and S789) is accompanied by a conformational change that partially dissociates caldesmon from actin (Kordowska et al., 2006). Such a structural change in H-caldesmon exposes the myosin-binding sites on the actin surface and allows actomyosin interactions in smooth muscles (Kordowska et al., 2006). In the case of non-muscle cells, the change in L-caldesmon weakens the stability of the actin filament and facilitates its disassembly (Kordowska et al., 2006). ERK-mediated phosphorylation of caldesmon has been shown to reverse the inhibitory effect of caldesmon on Arp2/3-mediated actin polymerization (Hai and Gu, 2006). The Arp2/3 complex is essential for podosome assembly, which are cytoskeletal adhesion structures that are important for cell invasion and extracellular matrix remodeling (Eves et al., 2006; Morita et al., 2007). Caldesmon is thought to be phosphorylated by ERK during the formation of podosomes (Hai and Gu, 2006). P21-activated kinase (PAK) is emerging as a major regulator of caldesmon-mediated actin dynamics *in vivo* (Foster et al., 2000; Lin et al., 2009). Reversible caldesmon phosphorylation at PAK-responsive sites is required for normal cell migration and cytokinesis (Lin et al., 2009). PAK phosphorylation sites (Ser-657 and Ser-687) are located close to calmodulin-binding sites (Mayanagi and Sobue, 2011). When caldesmon is phosphorylated by PAK, the ability to bind calmodulin is reduced by approximately 10-fold, and the affinity for actin-tropomyosin and the inhibition of actin-activated myosin ATPase activity are significantly reduced (Mayanagi and Sobue, 2011).

In addition, as one type of novel discovered posttranslational modification, lysine succinylation has been proven to be essential for regulating molecular functions, such as cellular metabolism, in physiological and pathophysiological states (Hirschey and Zhao, 2015). Caldesmon (lysine succinylation position 569) was downregulated in gastric cancer by LC-MS/MS analysis and validated by Western blotting (Song et al., 2017). Lysine succinylation position 569 of caldesmon may function as a potential biomarker in gastric cancer (Song et al., 2017).

## Clinical Applications of Caldesmon in Cancer

Alterations of caldesmon expression level in different types of cancers in the clinic have been investigated (summarized in the Table 1). The upregulated expression of caldesmon is generally observed in different cancers. However, downregulated expression of caldesmon is found in the blood vessels of malignant melanomas compared with both benign melanocytic tumors and normal tissues.

**TABLE 1:** Different expression trends of caldesmon isoforms in cancer.

Cancer types	Patient numbers	Isoforms	Tissue analyzed	Expression in cancer	Methods	Validations	Referencess
Glioma	87	L-caldesmon	Serum	↑	ELISA	IP, WB	Zheng et al., 2005
Colorectal cancer	38	L-caldesmon	Primary colon cancer and liver metastasis tissues	↑	2-DE, MS	WB	Kim et al., 2012
Gastrointestinal stromal tumor	105	H-caldesmon	Whole tissue	↑	IHC	/	Yu et al., 2019
Ovarian adult granulosa cell tumor	63	H-caldesmon	Whole tissue	↑	IHC	/	Yu and Qu, 2018
Epithelioid pleural mesothelioma	140	H-caldesmon	Whole tissue	↑	IHC	/	Comin et al., 2006
Oral cavity squamous cell carcinoma	155	L-caldesmon	Primary and metastatic tumor cells	↑	RT-PCR, WB	IHC	Chang et al., 2013
Oral cavity squamous cell carcinoma	292	L-caldesmon	Serum	↑	ELISA	/	Chang et al., 2013
Bladder cancer	18	L-caldesmon	Whole tissue	↑	AbM	IHC	Lee et al., 2015
Melanoma	79	H-caldesmon	The blood vessels within melanoma lesions	↓	IHC	/	Koganehira et al., 2003
Leiomyosarcoma	29	H-caldesmon	Whole tissue	↑	IHC	/	Watanabe et al., 2000
Fibroxanthoma	13	H-caldesmon	Whole tissue	↑	IHC	/	Martinez-Ciarpaglini et al., 2018

*ELISA, Enzyme-linked immunosorbent assay; IP, Immunoprecipitation; WB, Western blot; 2-DE, Two-dimensional electrophoresis; MS, Mass spectrometry; IHC, Immunohistochemistry; RT-PCR, Real-time quantitative polymerase chain reaction; AbM, Antibody microarray profiling. ↑, upregulated expression; ↓, downregulated expression; /, no validation.*

### Caldesmon as a Biomarker for Cancer

Caldesmon is important for the diagnosis of myoma (Rizzello et al., 2017). H-caldesmon is a highly sensitive and specific marker that shows smooth muscle differentiation and helps identify uterine mesenchymal tumors (Nucci et al., 2001; Saraydaroglu et al., 2008). It is reported that H-caldesmon is negative in normal endometrial stroma (0%, 0 case/25 cases) and endometrial stromal neoplasms (0%, 0 case/24 cases) (Nucci et al., 2001). In contrast, desmin is expressed in endometria (32%, 8 cases/25 cases) and endometrial stromal neoplasms (50%, 12 cases/24 cases) (Nucci et al., 2001). SMA (smooth muscle actin), the other markers of smooth muscle cells, is positive in endometrial stromal sarcoma (44%, 7 cases/16 cases) (Chu et al., 2001). Therefore, H-caldesmon can effectively distinguish endometrial stromal tumors from uterine smooth tumors. However, H-caldesmon is found expressed in some non-myogenic tumors, such as gastrointestinal stromal tumors, malignant pleural mesothelioma, and ovarian adult granulosa cell tumors (Comin et al., 2006; Yu and Qu, 2018; Yu et al., 2019). Therefore, H-caldesmon expression may not be conclusive evidence of myogenic differentiation, and the diagnosis should be referred together with other markers (Yu et al., 2019). In addition, L-caldesmon is also considered a potential serum marker for glioma (Zheng et al., 2005). Taken together, the different isoforms of caldesmon can be promising biomarkers for diagnosis and prognosis prediction.

### Mechanism of Caldesmon in Cancer Metastasis

#### Caldesmon Suppresses Podosome Formation

L-caldesmon is an integral part of the actin-rich core of the podosome (Eves et al., 2006). Caldesmon can suppress cell invasion by regulating the podosome/invadopodium formation of transformed and cancer cells (Yoshio et al., 2007). The overexpression of L-caldesmon suppresses podosome formation, whereas siRNA knockdown of L-caldesmon facilitates its formation (Eves et al., 2006; Gu et al., 2007). By analyzing the relationship between the expression levels of caldesmon and podosome/invadopodium formation in rat fibroblast (3Y1), RSV-transformed 3Y1 (BY1), human colon carcinoma (HCA7), murine melanoma (B16F10), human breast cancer (MB435s), and rat breast cancer (MTC) cell lines, podosome/invadopodium formation increases in transformed and cancer cells when caldesmon is expressed at low levels, and higher levels of caldesmon inhibit their formation (Yoshio et al., 2007). Caldesmon’s decreased expression has been identified in gastric cancer lymph node metastatic cells using a proteomics approach and loss of caldesmon expression could be associated with gastric cancer metastasis progression (Hou et al., 2013). In prostate cancer cells, a twofold increase in migratory capability and a threefold increase in invasion capability were found by scratch and Matrigel invasion assays after the knockdown of L-caldesmon expression (Dierks et al., 2015).

#### Caldesmon and Vessel Invasion

The presence of vessel invasion is considered indicative of a poor prognosis in many malignant tumors (Ekinci et al., 2018). Vascular smooth muscles contain both H-caldesmon (>75%) and L-caldesmon (<25%) (Glukhova et al., 1988). H-caldesmon appears to be the most specific and sensitive marker for vessel wall detection (Ekinci et al., 2018). The structural integrity and functional maturity of blood vessels are determined by the presence of normally functioning endothelial cells as well as the involvement of interendothelial junctions and mural cells (smooth muscle cells or pericytes) (Zheng et al., 2009). The knockdown of caldesmon caused serious defects in vasculogenesis and angiogenesis in zebrafish morphants, and the vascular integrity and blood circulation were concomitantly impaired (Zheng et al., 2009). The level of H-caldesmon expression in the melanoma blood vessels was inversely correlated with the frequency of metastasis (Koganehira et al., 2003). The endothelial cells of blood vessels in melanoma lesions appeared to be fragile compared to the normal tissues under electron microscopy (Koganehira et al., 2003). The fragility of blood vessels may increase metastasis.

### Caldesmon Decreases Chemoradiotherapy Susceptibility

L-caldesmon can decrease the chemoradiotherapy susceptibility of cancer cells. L-caldesmon-positive human colon cancer cell lines were more resistant to 5-fluorouracil (5-FU) and radiation treatment than L-caldesmon-negative cell lines (Kim et al., 2012). The expression level of L-caldesmon is therefore helpful in predicting the response of upper gastrointestinal carcinomas to neoadjuvant chemotherapy (Kim et al., 2012).

## Caldesmon and the Contraction of Intestinal Smooth Muscle

In addition, caldesmon can reversibly and cooperatively inhibit myosin binding actin to regulate smooth muscle contraction (Sobue et al., 1982; Ngai and Walsh, 1984). Smooth muscle dysmotility is the main pathogenic driver of gastrointestinal motility disorders. H-caldesmon can affect the contraction and relaxation of intestinal smooth muscle by binding to Ca^2+^/calmodulin and via phosphorylation (Wang, 2001). Structurally, H-caldesmon tethers myosin filaments to actin filaments to maintain the orderly arrangement of the thick and thin filaments. Functionally, H-caldesmon, as a “molecular brake,” sterically blocks actomyosin interactions in the resting state to modulate the development of contractile force (Guo et al., 2013). The expression of caldesmon was to be downregulated in rat models of chronic gastrointestinal motility hypofunction (Wang et al., 2001). Disruption of the normal inhibitory function of H-caldesmon could enhance intestinal peristalsis in both wild-type zebrafish larvae and mutant larvae that lack enteric nerves (Abrams et al., 2012). The detection of H-caldesmon phosphorylation sites by phosphorylation site-specific antibodies in colonic smooth muscle showed that H-caldesmon phosphorylation occurred on Ser-789 (Hedges et al., 2000). Ser-789 is phosphorylated by activated ERK, resulting in the C-terminal portion of H-caldesmon dissociating from actin and releasing the inhibition of ATPase activity, resulting in muscle contraction (Somara and Bitar, 2008). Expression levels of caldesmon in the gastric antrum were negatively correlated to gastric motility in rats treated by electroacupuncture (Yang et al., 2014). Expression of caldesmon was upregulated when gastrointestinal motility was inhibited. On the contrary, expression of caldesmon was downregulated when gastrointestinal motility was promoted (Yang et al., 2014). At present, the evidence correlating caldesmon and gastrointestinal motility disorders is not sufficient. However, whether caldesmon can regulate the contraction and relaxation of intestinal smooth muscle to treat gastrointestinal motility disorders needs further study.

## Conclusion

The biochemical features of caldesmon and its clinical implications in cancer have been reviewed in this article. The following main points are noted: (1) Alternative splicing of the caldesmon gene determines its different structures and the expression of its isoforms. (2) H-caldesmon and L-caldesmon conserve the completely identical sequences in the N- and C-terminal domains, and the central repeating sequence of H-caldesmon is deleted in L-caldesmon. (3) Although H-caldesmon and L-caldesmon have similar functional domains, their tissue and cell distributions are different. (4) Caldesmon can be a biomarker for the pathological diagnosis of tumors and the prediction of chemoradiotherapy response. (5) Caldesmon can suppress tumor metastasis by regulating podosome/invadopodium formation and vasculogenesis. Future research aspects may include (1) clinical data about the relationship between expression of the two isoforms in cancers (primary and metastasis) and patient survival; (2) the effects of the expression of upregulated or downregulated isoform in cancers (primary and metastasis) on cell motility and invasive characteristics; and (3) evidence-based clinical studies or animal models on the role of caldesmon in gastrointestinal motility disorders are critically required.

## Author Contributions

Y-BY and C-FX prepared literature research and wrote the manuscript. J-GL conceived the ideas and wrote the manuscript. CW and Y-BY designed project, wrote the manuscript, and prepared figures. All authors contributed to the article and approved the submitted version.

## Conflict of Interest

The authors declare that the research was conducted in the absence of any commercial or financial relationships that could be construed as a potential conflict of interest.
